# Sex matters in preclinical research

**DOI:** 10.1242/dmm.050759

**Published:** 2024-03-07

**Authors:** Monica J. Justice

**Affiliations:** Program in Genetics and Genome Biology, The Hospital for Sick Children, Toronto, Ontario, M5G 0A4, Canada

**Keywords:** Biological sex, Preclinical research, Sexual dimorphism

## Abstract

International Women's Day 2024 has a theme of inclusion. As publishers of preclinical research, we aim to show how inclusion of females in research advances scientific rigor and improves treatment reliability. Sexual reproduction is key to all life across the plant and animal kingdoms. Biological sex takes many forms that are morphologically differentiated during development: stamens versus pistils in plants; color and plumage in birds; fallopian tubes versus vas deferens in mammals; and differences in size, for instance, males are smaller in the fruit fly *Drosophila melanogaster*. Physical differences may be obvious, but many traits may be more obscure, including hormonal, physiological and metabolic factors. These traits have a big influence on disease and responses to treatment. Thus, we call for improved inclusion, analysis and reporting of sex as a biological variable in preclinical animal modeling research.

## Teaser

**Summary:** To mirror the theme of inclusion for International Women's Day 2024, we stress the need for improved inclusion of sex as a biological variable in preclinical research.

## The importance of including both sexes in research

The history of including women in research studies is fairly recent. Inclusion of women in research studies was not promoted by the National Institutes of Health (NIH) in the USA until 1986, so male organisms and cells were primarily used in research. Prior to 1993, the US Food and Drug Administration (FDA) prohibited women of childbearing age to be included in clinical trials; thus, men were the primary subjects. In 2009, the Canada Institutes for Health Research (CIHR) implemented a policy that required researchers to explain their choice for inclusion or exclusion of both sexes. This policy has now been updated to include gender issues as a priority area. In 2012, the European Commission funded nine national agencies across Europe, designed to enhance gender and sex equality in research as they mandated the report of gender- and sex-based analyses in scientific communications (Heidari et al., 2016). Finally in 2014, the NIH issued a mandate to include sex and gender as biological variables in cells, tissues, organisms and cell lines in all studies that were awarded grants, unless biological sex was not relevant as shown by rigorous logic and analysis (Clayton and Collins, 2014). But funding agencies should not be alone; institutional human and animal review committees, publishers and reviewers should be rigorous in requiring the inclusion of both sexes in preclinical research (Zakiniaeiz et al., 2016).

At Disease Models & Mechanisms (DMM), our goal is to connect basic and applied science, aiming to publish work that supports preclinical research in many organisms. Supporting robust standards for preclinical testing of animal models drives reproducibility and translatability in research**.** To that end, DMM asks authors to follow best practice guidelines regarding experimental subjects, data reporting and statistics. As expressed by the Animal Research: Reporting of *In Vivo* Experiments (ARRIVE) guidelines (Kilkenny et al., 2010; Percie du Sert et al., 2020), appropriate and transparent reporting and representation of methodological procedures and data are essential for preclinical study interpretation and translatability. To fully adhere to these guidelines, sex, defined here as the biological genetic criterion (Box 1), should be reported and analysed as a biological variable (Song et al., 2023).

**Figure:**
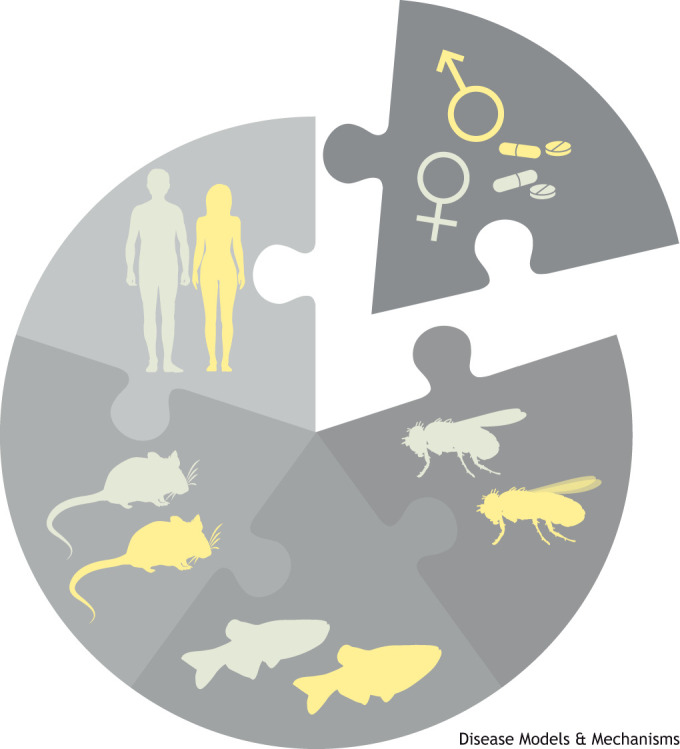

**Box 1. The genetics of sex**

Research organisms have many ways of determining sex. The roundworm *Caenorhabditis elegans* can be a hermaphrodite (female) or male based on the ratio of sex chromosomes to other chromosomes (autosomes) (Farboud et al., 2013). Fruit flies (*Drosophila melanogaster*) have X and Y chromosomes, and sex is determined by the X chromosome ‘dose’ in a manner distinct from that of worms (Erickson and Quintero, 2007). Other insects have many unique ways to determine sex, including losing the paternal genome entirely. Fishes, reptiles and amphibians have a multitude of methods, many of which include environmental cues, such as temperature or social environment (Marshall Graves, 2008). The sex-determining genes in zebrafish (*Danio rerio*) have not yet been identified. Female placental mammals have two X chromosomes, whereas males are the heterogametic sex with an X and a Y chromosome, which contains the mammalian sex-determining gene *SRY* (Koopman et al., 1991). In female mice, gene expression on the X chromosome is modified by epigenetic programming that inactivates the X chromosome in an ‘imprinted’ way (Lee and Bartolomei, 2013). In mammals, regions of the autosomes also undergo imprinting, depending upon whether the chromosome is inherited from the mother or the father, conferring autosomal epigenetic differences in gene expression. Certainly, each of these mechanisms affect the expression of genes. With a multitude of ways to determine the sexes, it is no wonder that sex is an important and distinguishing variable that plays a big role in interpreting disease phenotypes and responses to therapies.

This is imperative because men and women are affected by different diseases, develop the same diseases in different ways and have different responses to drugs. Some of the leading causes of death in the world show differing incidences and presentation between men and women, including heart disease and stroke, metabolic syndrome, cancer and neurodegenerative diseases (summarised in Austad and Fischer, 2016). Although the incidence of most diseases causing death, including cancer and heart disease, is more predominant in men, the degree of symptom severity and responses to treatment can also vary widely between the sexes. Furthermore, many aspects of the central nervous system differ between men and women, as the brain responds to the sex hormones, androgen, estrogen and progesterone. Gene expression is highly regulated by the binding of estrogen to its receptors in the brain, and their subsequent translocation to the nucleus, where estrogen-responsive elements modulate gene expression. Genotype and sex can cause many different manifestations of neurodegenerative disease in men and women, often due to genetic polymorphisms that affect a limited number of genes (summarised in Gamache et al., 2020). For instance, women are more likely than men to have the neurodegenerative Alzheimer's disease (Fisher et al., 2018). Other neurodegenerative diseases, including multiple sclerosis, Parkinson's disease, dementia with Lewy bodies (DLB) and amyotrophic lateral sclerosis (ALS), show sex differences as well. Women are more likely to have multiple sclerosis, whereas Parkinson's disease, DLB and ALS are more prevalent in men.

Treatments, too, can affect one sex differently than the other. In the past, the exclusion of female animals from many preclinical trials led to failures in the clinic. Perhaps the most tragic failure was the use of thalidomide to prevent morning sickness during pregnancy, resulting in a multitude of congenital abnormalities. For many drugs, women are more sensitive, making dosing an issue in efficacy. Some drugs have an adverse effect in women and others show a response only in women (as described in a report to the US Congress by Janet Heinrich; Zucker and Prendergast, 2020). Still, many preclinical studies have used males primarily, in spite of the intended use of the drugs in women.

Rodents are very often used as preclinical models, yet in 1990 through to 2009, 80% of all mouse/rat studies used males exclusively (Beery and Zucker, 2011). Not reporting the sex of animals studied, examining one sex or combining data from both sexes can lead to misleading results, particularly in preclinical studies. Not including sex in data analysis can also lead to loss of information and misinterpretation of data. So, why is sex not always reported?

Arguments against the use of both sexes in preclinical research include the difficulty in controlling hormonal fluctuations in females, reducing animal usage, which decreases time, space used and cost, and even protective or paternalistic views of women. In mice, females are required as breeders, making males the surplus animals that are often used in experiments. On the other hand, female mice are more easily group housed than males, which often battle each other, making females a preferred sex in experiments that require housing the mice for long periods of time, such as in cancer and ageing experiments. Determining the sex of each animal can also be challenging: for most mammals, a sexually dimorphic appearance is apparent when the animals are adults, but sex can be difficult to detect in pre-pubescent or embryonic stages. Examining the sex of mammalian embryos requires researchers to determine the presence of the Y chromosome in each conceptus. For flies and fish, a careful examination of each organism is required, sometimes after the experiment is carried out. Therefore, an experiment must be carefully planned and requires more work to include both sexes adequately. Here, we show an improvement in the inclusion of both sexes in preclinical studies, while suggesting that further improvements can be made in the inclusion and reporting of both sexes to enhance the transparency and interpretation of research results.

## Sex-specific effects in model organism phenotype

Fruit flies are the most studied experimental genetic organism and 30% of genes show sex-specific biases in adult gene expression (Parisi et al., 2004); thus, sex-specific differences are expected. Male fruit flies are smaller than females, and even larvae show sex differences in size. Furthermore, the neural circuits develop in a sexually dimorphic way, leading to sex differences in behaviour (Sato and Yamamoto, 2023). In mammals and fish, differences in gene expression can translate into differences in small molecules such as hormones, hormone-responsive elements and, therefore, many downstream signals that can affect multiple organ systems, including kidneys, heart, blood, bones and the nervous system. These differences can translate into a multitude of sex-specific effects, including a larger effect in one sex, a phenotype in only one sex, or opposite phenotypes in the two sexes.

The International Mouse Phenotyping Consortium (IMPC) was designed to make knockout alleles of every gene in the mouse genome and carry out a standardised phenotyping platform designed to uncover clinically relevant traits (Birling et al., 2021). Ten phenotyping centres in the IMPC examined 234 traits in wild-type mice, to find that sex alone could explain a difference in nearly 31% of 1448 data sets examined. By analysing phenotypes in 2186 strains of knockout mice, 17% of the phenotypes were found only in one sex or were opposite in the sexes (Karp et al., 2017). In cases where the phenotypes were opposite in the different sexes, not analysing the data separately would have made the trait seem normal. As these data were obtained from a high-throughput project, the data might have shown additional differences if they had been obtained with cohort sizes designed to statistically parse out sex differences.

In recent years, there have been a growing number of studies focused on specific areas of disease that are considering sex variability in study design and analysis. Beaudry and Law (2023) extracted data from reports of a single gastrointestinal adenocarcinoma cachexia model that had been used in 246 publications over a 30 year period to find that 76% of the studies used males only, 16% used females only, only 2% used both sexes and 6% (or 14) of the studies (of which 11 occurred after the ARRIVE guidelines were published) failed to report the sex of the animals at all. Although cachexia, or wasting and weight loss, is often a lethal accompaniment to cancer, the authors found that many of these publications did not report standards such as body weight versus tumour weight, so they conducted experiments in their own laboratory that used both sexes. In the sex-compared study, females did not show as much body wasting as males (Beaudry and Law, 2023), perhaps explaining why males were used so frequently, but raising caution as to extrapolating to females any treatments that might be tested in the model. The study highlights the importance of proper reporting for any cancer-modelling study and shows the dangers of using historical data sets for comparison with treatment paradigms.

Many cancers, other than those associated with a single sex, such as prostate and breast cancer, may have a different predominance or outcome in one sex or the other, with an overall increased incidence (Austad and Fischer, 2016). Using zebrafish as a model, Montal et al. (2023) studied the incidence and progression of melanoma in obese fish. Male obese fish had an increased incidence and melanotic tumour burden compared to that in females. Although obesity is considered to be a risk factor for the invasiveness of melanoma in males, this study is the first to reveal a sex-specific effect, confirming clinical findings in humans.

Sex differences are also seen in obesity and metabolic diseases, which often have an inflammatory component. It is more difficult to induce obesity in female rodents, including mice and rats, than in males. Talley et al. (2022) used an inflammation-reporter mouse model to sensitively detect inflammasome production in obese mice when placed on a high-fat diet. In this study, inflammatory pathways were more pronounced in males, who also developed metabolic syndrome earlier. It appears, as in humans, that estrogen has a protective effect against insulin resistance early in metabolic disease (Eid and Feldman, 2021).

Sex hormones are well known to affect immune responses (Gubbels Bupp, 2015) and many autoimmune diseases are more prevalent in women. Using a mouse diabetes model, Markle et al. (2013) showed that the microbiome could alter sex hormone differences and the occurrence of autoimmune-mediated type I diabetes in female mice. Strikingly, changing the microbiome to an adult male profile could change the occurrence of autoimmune disease and, thus, prevent type I diabetes. Thus, the microbiota can produce metabolites that modulate the occurrence of inflammation in a sex-dependent manner.

Peripheral neuropathy is a painful consequence of metabolic syndrome in patients with type 1 or type 2 diabetes, which also presents differently in females and males. However, in three different mouse and rat models of type 2 diabetes, peripheral neuropathy developed equally in males and females, and both had similar neuronal gene expression and lipid profiles that showed inflammation and altered lipids, despite a later onset of insulin resistance in females compared to that in males (Elzinga et al., 2021; O'Brien et al., 2018). Thus, assumptions about accompanying phenotypes in disease conditions may be misled if females and males are not considered separately.

People with variants in connexin 30 (GJB6) often present with hearing and skin anomalies, but this transmembrane protein is also known to be expressed in ependymal cells in the brain. Novielli-Kuntz et al. (2021) engineered mice with a pathological connexin 30 mutation that dampened cognitive function, but exclusively in females. This points to potential sex-related differences that should be considered in humans with connexin 30 variants and highlights fundamental sex-dependent differences in the brain.

As mentioned above, several neurological conditions present differently in males and females. Some mouse models of Alzheimer's disease reproduce the higher prevalence of the disease in females, as many exhibit more severe phenotypes in females than in males, but not all models are consistent with findings in humans (Sanfeliu et al., 2024). A series of nine pathogenic mutations in valosin-containing protein (VCP), which is associated with neurodegenerative diseases including ALS, were generated by CRISPR/Cas9 in the fruit fly, and both sexes were examined in each case (Wall et al., 2021). Unexpected differences were found in mitochondrial respiration in males only, consistent with the observation that age-related symptoms were less severe in female flies. This suggests that VCP disease mutations could vary greatly in severity, informing human disease mechanism.

Overall, these studies demonstrate that not all sex-dependent effects in model organisms of disease will be reflected in humans, and some sex-dependent effects of disease in humans will not be recapitulated in all model organisms, as demonstrated in some of the Alzheimer's disease mouse models. However, knowing and documenting these differences will improve the validity of disease models (Justice and Dhillon, 2016). It may also reveal unexpected sex differences in models of patient-derived mutations, as exemplified by the introduction of variants in VCP and connexin 30 discussed above. In any case, diligently including both sexes in all preclinical studies and examining disease in multiple model systems will inevitably improve reliable translation of research to benefit both men and women.

## Differences in treatment responses

Examining both sexes in preclinical animal models could provide evidence for sex-specific responses to drugs that will help prevent failures in clinic. Xanthohumol, a prenylated flavonoid found in hops, improves cognition in mice fed a high-fat diet, and has thus been examined as a possible treatment for cognitive decline in Alzheimer's disease. Using mice, Kundu et al. (2022) showed that xanthohumol improved cognitive effects in female mice alone, which was due to increased levels of healthy fats in the hippocampus, which were not increased in males. These data show that xanthohumol may have a therapeutic effect on cognition, regardless of diet, and perhaps specifically in women. It is clear that the therapeutic potential of this drug would have been missed if only male mice were used in this study.

Persistent activation of the P2X7 (or P2RX7) receptor causes neuroinflammation and progresses disease in certain mouse models of ALS. Treating male and female mice with an antagonist of this receptor showed delayed disease onset and altered progression in female mice only, with no beneficial effects in males (Ruiz-Ruiz et al., 2020). Furthermore, in neuropharmacology studies, differences in behaviour are commonly found between the sexes in zebrafish, suggesting that fish may play an important role in developing treatment strategies for neurological disease (Genario et al., 2020). Rats can also be used in behavioural studies and Simpson et al. (2012) showed many sex-specific differences in response to mood-altering drugs in rats. The authors concluded that all behavioural analyses should be designed to detect sex differences. Certainly, the inclusion of both sexes in all preclinical studies would avoid false negatives in drug discovery pipelines, uncover any sex-specific adverse effects and ultimately save time and money when advancing to clinical trials.

## Implementing sex as a biological variable

Segregating data by sex should be common practice for researchers. Data should be analysed separately prior to pooling the data, to prevent ‘hiding’ sex differences. In any experiment, sexes should be randomised, balanced and recorded in experimental and control groups. Finding no sex difference in data is also an important result that should be reported to inform and support future research. To reduce animal usage, a pilot study can be conducted to determine whether a larger study that includes sex as a biological variable is warranted.

Sex should also be reported for cell lines and organoids. Gene-editing technology tends to target each locus differently, sometimes creating heterozygous mutations. Knowing that a cell line is female and targeting both copies on the X chromosome is required for valid results to be obtained for an X-linked locus.

Sex is only one of the standards that must be met to ensure translatability in preclinical studies, while ensuring best practices in animal research (Justice and Dhillon, 2016). Nevertheless, it is the role of animal care committees, journals and reviewers to ensure that sex as a biological variable is reported. An international set of guidelines for implementing and reporting sex as a biological variable was published by the Gender Policy Committee of the European Association of Science Editors (EASE) in 2016 (Heidari et al., 2016). These Sex and Gender Equity in Research (SAGER) guidelines are a comprehensive procedure for reporting sex and gender information in study design, data analyses, results and interpretation of findings. The goal is to establish a systematic approach to the reporting of sex and gender in research across all disciplines. At DMM, we encourage authors to adhere to the SAGER guidelines and refer to the SAGER checklist when submitting their research to us.

Including females in preclinical research has been a big step towards improving the health of women. However, 10 years after the implementation of sex as a biological variable by the NIH, improvements can still be made, as we have documented here. Raising experimental standards to report sex as a biological variable has revealed many hidden differences between the sexes. Now, determining the underlying mechanistic basis for sex differences in phenotype or responses to drugs could reveal unchartered research territory that can be addressed in model organisms. Supporting the theme of International Women's Day, inclusion of females in preclinical research should become an unconscious standard practice for researchers, which will improve our understanding of diseases and treatments, and ultimately improve the health of all people, regardless of biological sex or gender identity.
